# The concept of RNA-assisted protein folding: the role of tRNA

**DOI:** 10.1186/1742-4682-9-10

**Published:** 2012-04-02

**Authors:** Jan C Biro

**Affiliations:** 1 Karolinska Institute, Stockholm, Sweden; 2 Homulus Foundation, Los Angeles, CA, USA

## Abstract

We suggest that tRNA actively participates in the transfer of 3D information from mRNA to peptides - in addition to its well-known, "classical" role of translating the 3-letter RNA codes into the one letter protein code. The tRNA molecule displays a series of thermodynamically favored configurations during translation, a movement which places the codon and coded amino acids in proximity to each other and make physical contact between some amino acids and their codons possible. This specific codon-amino acid interaction of some selected amino acids is necessary for the transfer of spatial information from mRNA to coded proteins, and is known as RNA-assisted protein folding.

## Background

The concept of nucleic acid-assisted protein folding (nucleic acid chaperons) was first published in 2005 [1], when it was suggested that mRNAs participate in the formation of the tertiary structure of their coded proteins; this function is in addition to the well-recognized determination of the amino acid sequence of the proteins, as described in Nirenberg's Genetic Code. Messenger RNAs often have a tertiary structure that provides spatial information for protein folding, i.e. they are nucleic acid chaperons [2]. Nucleic acid-assisted protein folding requires that mRNA and coded peptides remain in contact with each other, even after the polymerization of the amino acids during translation. The original model described RNA-assisted protein folding, but the role of tRNA was not properly elucidated.

The role of the t-RNAs is to "translate" between mRNA and the encoded peptide, which means that tRNAs recognizes the codons in mRNA and arrange the amino acids in the order of the codons for polymerization by the protein synthetase. This function could be performed by a much smaller oligonucleotide containing only four nucleotides, three for the anticodon and one for binding the corresponding amino acid. However tRNAs are much larger, about 76 nucleotides in length.

The abundance of tRNA is another mystery. The 64 different codons require 64 different "mediators", but there are many more. There are, for example, 359 different cytoplasmic tRNAs in human cells, and many more when genomic variants are included. The generous size and lavish redundancy suggest that the function of tRNAs is far more complex than the passive connecting of codons with their amino acids that has been recognized to date.

The aim of this work is to identify additional functions for tRNAs in translation, and to explain their involvement and role in nucleic acid-mediated protein folding.

## Results and discussion

The existence of tRNA was hypothesized by Francis Crick in 1956 [3,4] to explain the transfer of information (translation) from nucleic acids to proteins, which are two structurally very different molecules. The first tRNA sequences were described and structures were suggested in 1965 by Holley *et al*. [5]. Transfer RNA was required to "fill the gap" between amino acids and codons, or spatially "convert" the large codons (3 nucleotides, ~1 nm in length) to amino acids (single molecules, about 1/3 nm length when incorporated in proteins).

**Figure 1:**
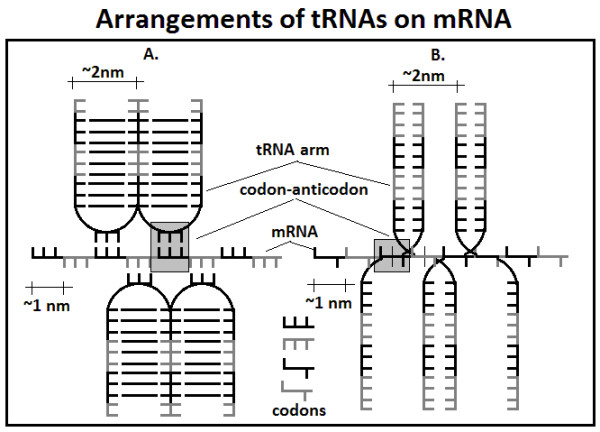
**Alternative arrangements of tRNAs on mRNA**. The diameter of the arms of tRNA in the classical cloverleaf structure is approximately 2 nm, double the distance between codons (1 nm). Side-by-side arrangement is not possible, only for every 2nd tRNA in this form (A). The unfolded (linear) structure permits a side-by-side arrangement for every tRNA as well as extensive complementary interactions between them (B).

The tRNA seemingly solves the problem because of the 3:1 size difference between codons and amino acids (i.e. mRNA and the coded peptide). However, this creates a new spatial problem: the tRNA molecules are far too large to be able to align side-by-side (codon after codon) on the mRNA. Transfer RNAs are typically 73-93 nucleotides in length, and they have a highly conserved characteristic tertiary structure in which the amino acid acceptor CCA tail and the anticodon are located at opposite ends of the molecule, about 7.6 nm from each other. The molecule has three arms with loops (called D, T and anticodon loops), together with one additional, variable loop. The L-shaped structure fits into a 6 × 6 × 2 nm cube. The thickness of the tRNA (approximately 2 nm in a cloverleaf structure) is twice that of the distance between codons (1 nm). Consequently, only every 2^nd ^folded (cloverleaf) tRNA would be able to approach the mRNA from the same direction. Alternatively, the tRNA could unfold into a linear single-stranded form (with a diameter corresponding to the distance of 1 nm between codons). However, the unfolded, linear form would interact with its neighbor (Figure 1).

The structural complexity of tRNA is reminiscent of that of a protein, with 71 out of 76 bases participating in a stacking interaction (42 of the bases have a double helical stem structure). A series of specific 9 bp sequences crosslink the tertiary structure of the tRNA by interacting with bases from a different stem and loop region of the molecule. All of these 9-bp interactions are non-Watson-Crick associations and are highly conserved, which makes it likely that all tRNA molecules have similar structures, although only a few have been crystallized and their structures determined.

To provide a one-to-one correspondence between tRNA molecules and the codons that specify amino acids, 61 types of tRNA molecules would be required per cell. However, many cells contain fewer than the predicted 61 types of tRNAs because the wobble base is capable of binding to several, though not necessarily all, of the codons that specify a particular amino acid. A minimum of 31 tRNAs are required to translate, unambiguously, all 61 sense codons of the standard genetic code [6,7].

However, the total number of tRNAs is much larger than the number of codons. In the human genome, which according to estimates has about 27,161 genes [8], there are in total about 4,421 non-coding RNA genes, which include tRNA genes. There are 22 mitochondrial tRNA genes, 497 nuclear genes encoding cytoplasmic tRNA molecules, and 324 tRNA-derived putative pseudogenes [9].

This means that there are, on average, eight slightly different cytoplasmic tRNAs for every possible codon. Therefore, the size and the redundancy of tRNAs are very large for the "tiny" function that is accepted today. Identifying new functions and explaining the size and redundancy of tRNA is one of the goals of our study.

The tRNA database that we used [10,11] lists 359 human tRNA sequences. There are tRNAs corresponding not only to the 20 amino acids, but also to the initiation (Ini, CAT, Met) and termination (Sec, TCA) sites. Many codons have no tRNA representations in this database, including AAA (Lys), ACA (Thr), ACC (Thr), ACT (Thr), ATC (Ile), ATG (Met), CTA (Leu), GAC (Asp), GAG (Glu), GCG (Ala), GGA (Gly), GGC (Gly), GGG (Gly), GGT (Gly), TTA (Leu). Each of the remaining 49 codons is represented by an average of 7-8 different tRNAs.

(The relatively large numbers of tRNAs may be readily explained by the fact, that many fundamentally important genes code isoforms of the same proteins (for example the enzymes of glycolysis), so that a mutation which result a loss of function will not be immediately lethal. In case of only one tRNA alternative, this would have been way too risky for life.)

It is important to keep in mind that almost all of these tRNAs have been identified using bioinformatics tools that were looking for sequence characteristics assigned to real tRNAs. However, their existence and biological functions have yet to be confirmed by biochemists. Even the Protein and Nucleic Acid Databases (PDB, NDB, [12,13]) are surprisingly empty of real tRNA structures, although many tRNA-associated proteins have been the subject of structural studies.

*When speaking about tRNA the reader has to keep in mind that translation is still a very "hot" subject for many scientists, "the dark side of molecular biology" *[14]*driven by a powerful paradigm. This 50 year old paradigm suggests that translation is a "tape reading" where there is a "tape" (mRNA) and step-by-step reading it (tRNA) provides proteins on the surface of ribosomes. The ribosome has a door "in" (A site) and a door "out" (P site) *[15,16]. *This "cartoon guide to translation" *[14]*very strictly separates the "RNA World" from the "Protein World", where the only connection permitted is the tRNA (the "adaptor"). However the connection between codons and amino acids (provided by tRNA) is not logical or the result of evolution, but accidental, a so called "frozen accident" *[17]*and codons and amino acids never interact with each other. Consequently, in every published structure, the distance between the amino acid and the anticodon is well over 50 Å*

*Although this mechanical model is supported by structural studies *[18-24]*the reality is that crystal structures present only snapshots of thermodynamically stable conformations. Solution and computational methods provide evidence for the inherent flexibility of tRNA structure under a variety of conditions and for differing tRNA species. Transfer RNAs perform a wide range of motion and conformational changes and allosteric transition during the process of translation*. [25-27]*Lapointe, Alexander, Caulfield]. Molecular Dynamic methods, like simulations of Cryo-EM Microscopy and X-Ray Data to explore intermediate conformational space, often provide previously unidentified structures which are very different from the canonical cloverleaf-like manifestations*. [27-30]. *Interaction with other macromolecules is known to cause structural alteration in tRNAs*. [31-33]. *The tRNA structure is sensitive for changes in the molecular environment, post-transcriptional modifications. A single base mutation is sufficient to change the "cloverleaf" configuration into a "hairpin"-like folding *[29,34].

An estimation of the folding energy (Quickfold program [35]) indicates that all human tRNAs have negative folding energies (-26.1 ± 4.42 kcal/mole, SD, n = 359). However, even randomized tRNA sequences possess significant folding energies (-21.1 ± 4.79 kcal/mole, SD, n = 369). The difference between these two values (-4.95 ± 5.66 kcal/mole, SD, n = 369), the Free Folding Energy, FFE, is rather small, only approximately 19% of the total folding energy, and there are even numerous positive values. This indicates that the majority of the total folding energy of tRNAs is provided by the nucleotide composition, and not by the nucleotide sequence itself, i.e. tRNAs may fold into numerous different structures in addition to the typical cloverleaf configuration.

**Figure 2:**
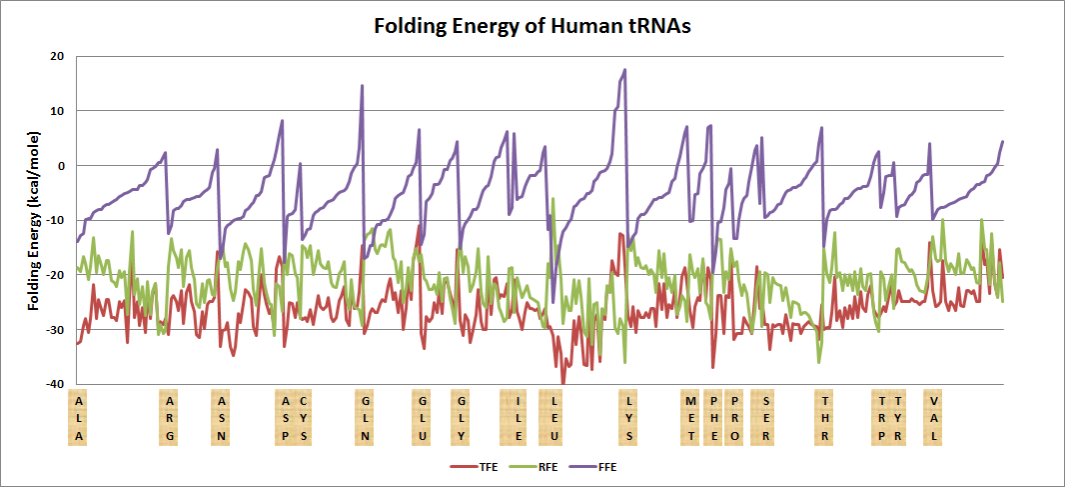
**Folding energy of human tRNAs**. Folding energies were determined for 359 different sequences and the values were sorted by amino acids and their increasing Free folding energy (FFE) values. The intact (total, TFE), randomized (RFE), and free (FFE) folding energies are plotted. FFE = TFE-RFE. Free energy estimates were determined using the Mfold and DINAMelt Web Servers [35,56-58].

**Figure 3:**
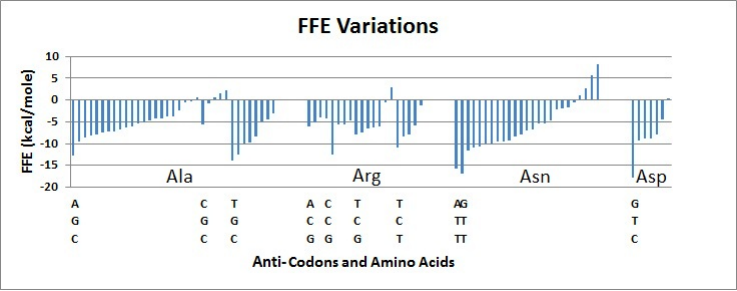
**FFE variations**. The FFEs of tRNAs for 10 different anticodons (encoding four amino acids) were sorted in ascending order and are indicated by the bars.

The total and free folding energies show wide variation among different tRNAs, even when these different tRNAs code for the same amino acids. It is not possible to correlate this variation to the codons either, because tRNAs carrying the same anticodon vary widely in the size of their folding energies (Figures 2 and 3).

The common feature of all tRNAs is that their primary structures contain a large number of complementary residues and these Watson-Crick-type interactions, within the same sequence, provide a characteristic tertiary structure. This property suggests that complementary interactions do exist not only within but also between tRNAs, as they have a high degree of similarity. The question of interactions between tRNAs was studied here using *in silico *and hybridization (melting) studies.

Interaction between tRNAs is thermodynamically favored. Not unexpectedly, the tRNA hybridizes with itself (dG values for intact and randomized sequences are -43.7 ± 8.22 kcal/mole, SD, n = 359 and -33.5 ± 6.76, SD, n = 359, respectively, and the difference, the Free Hybridization Energy, FHE, is -10.2 ± 7.18 kcal/mole, SD, n = 359). The codon- and amino acid-related distribution of dG values for hybridization is similar to the distribution of dG values for folding in that there is a wide range, and even some positive values; however, the correlation between FFE and FHE is not significant (data not shown). The mean dG of hybridization is much higher than the mean dG of folding, indicating that the potential for tRNA interactions (Watson-Crick pairs) is not fully utilized during folding compared to hybridization.

**Figure 4:**
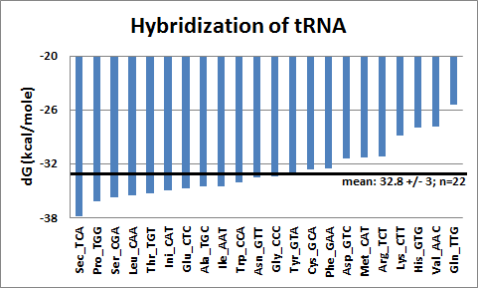
**Hybridization of Trna**. Each tRNA was hybridized to every other (359), and grouped according to the specific amino acid encoded. Each bar represents the mean of 359 hybridizations. The mean ± SD of the 22 groups is indicated by the horizontal line. (Ini: initiation codon, Sec: termination codon. Amino acids are listed with their anticodons). Free energy estimates were determined using the Mfold and DINAMelt Web Servers [35,56-58].

Hybridization between different tRNAs (359 vs. 359) in any combination is possible and is thermodynamically favored (Figure 4).

*The idea that tRNAs interact with each other during translation is not new. It was first suggested by Carl Woese in 1970 *[36]*in his "reciprocating ratchet mechanism" for protein synthesis which depends upon conformational changes in tRNA and allosteric transitions in place of translocation*.

**Figure 5:**
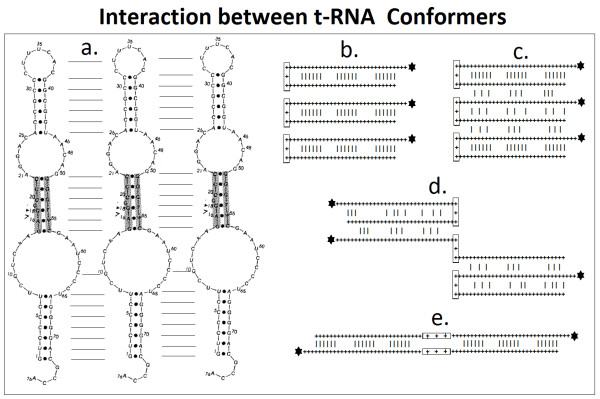
**Interaction between t-RNA Conformers**. There are many different forms (conformers) of t-RNAs in addition to the canonical "cloverleaf" configuration. The hairpin-like structure (a) is suitable for inter-molecular interactions (horizontal lines) in addition to the numerous intra-molecular Watson-Crick complementary contacts (dots). These interactions can lead to dimerization (b-e) of t-RNAs. A parallel alignment of 3 t-RNAs, for example, (b) de-stabilizes the intra-molecular interactions between bases in the middle t-RNA (c) and unfold it (d) or "zip it" into a dimer (e). The secondary structure of the E. coli tRNA Glu was adopted from [39] (Left). The schematic structure and interactions of tRNAs are depicted in the right side of the figure. The anticodons are indicated by the boxes and the amino acid by asterisks. The+ - +signs indicate complementary bases.

*The cloverleaf model, the paradigm of tRNA structure, emphasizes the high conservation of the base pairing pattern or secondary structure among tRNAs encoded in the genome of all organisms and eukaryotic organelles. However, some tRNAs are not able to fold into this patter *[37]. *Other tRNAs exists in more than one thermodynamically stable conformations (conformers) and these unconventional structures are in dynamic equilibrium with each other *[38]*and even interact with each other *[39]. *It is well recognized that sequence variations and post-transcriptional modifications can case large variations in the folding energy, half-life and activity of tRNAs *[40,41]. *There are many different tRNAs with the same anticodon, therefore it is reasonable to suppose that tRNAs are heterogeneous regarding their structure and thermodynamic properties *[42,43]. *Dimer formation by tRNAs under physiological conditions have been suggested *[44]*and modeled *[39]. *We suggest that the hairpin-like conformers of tRNAs are able to form complementary base interactions not only within bot even between identical or similar tRNAs and form homo- and heterodimers (Figure *5*)*.

### Model of tRNA structures and interactions

**Figure 6:**
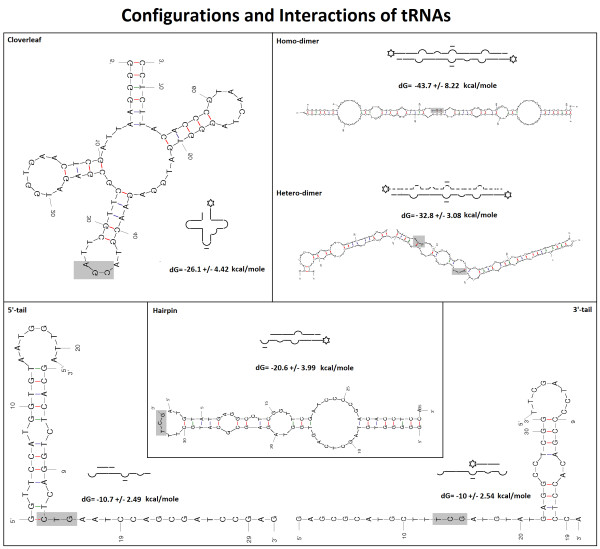
**Configurations and interactions of tRNAs**. Schematic representation of the main configurations of tRNAs with corresponding examples. The major turns (known from the cloverleaf form) are indicated by half-circles, the 3' amino acid by an asterisk, the codon site by underlining, and the anticodons by gray boxes. The inserted numbers are the means ± SD (n = 359) of the thermo-dynamic energies (dG) of the corresponding configurations.

The low FFE of tRNAs indicates that they may have more than one thermodynamically possible configuration. At the same time there is the potential for intermolecular interactions with other tRNAs. Some of the most frequent folding patterns and interactions are listed in Figure 6.

**Figure 7:**
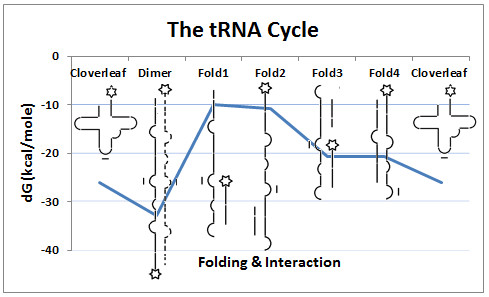
**The tRNA cycle**. The tRNA has many possible different foldings and interactions during translation, and the molecules pass through these stages, completing the tRNA cycle (from left to right). The corresponding folding and hybridization energies (dG) are indicated by the blue line. For more details see the text.

The cloverleaf folding is the best known form of tRNA structure. However, several alternative forms have been suggested [4] since 1965 when Holley *et al*. described the first sequence of a tRNA [5]. We would like to return to these structural variations because their simultaneous existence provides a more detailed understanding of the kinetics of translation. Notice the difference in the folding energies (dG) of the different forms. The following model is suggested (Figure 7):

1. The native, free, cytoplasmic tRNA has a cloverleaf structure, dG:~-26 kcal/mole.

2. The tRNAs come into intimate contact with each other in the mRNA-rRNA-tRNA complex. This proximity destabilizes the cloverleaf structure and transforms the molecules into thermodynamically more favored homo- and hetero-dimers (dG: ~-43.7 and ~-32.8 kcal/mole, respectively. p < 0.001). Statistical analyses were performed using Student's t-test [45].

3. However, the dimer is not stable either, because the arrival of a 3rd tRNA, which has affinity for the 2nd tRNA, provides competition with the 1st tRNA and destabilizes the bonds between the 1st and 2nd tRNAs in the first dimer.

4. The 1st tRNA is released from the first dimer and a 2nd dimer is formed by the 2nd and 3rd tRNAs.

5. The free 1st tRNA refolds into 5'-tail (dG:~-10.7 kcal/mole), 3'-tail (dG: ~-10 kcal/mole), double tail (5'- and 3' tails together dG: ~-20.7 kcal/mole), and hairpin (dG:~-20.6 kcal/mole) forms, and finally to the original cloverleaf structure (dG: ~-26.1 kcal/mole); the structural cycle is then completed.

6. The cycle repeats itself with the 2nd, 3rd ... etc. tRNAs as the translation process continues to its completion.

Dimerization or aggregation of naturally-occurring tRNAs *in vitro *is a known phenomenon, but typically occurs under non-physiological conditions [46-49]. Unmodified tRNA transcripts, as well as mature tRNAs, can undergo complex formation [48]. The dimeric tRNA forms are often associated with pathological conditions [50]. However, we suggest that dimerization of tRNA is a normal, natural phenomenon.

**Figure 8:**
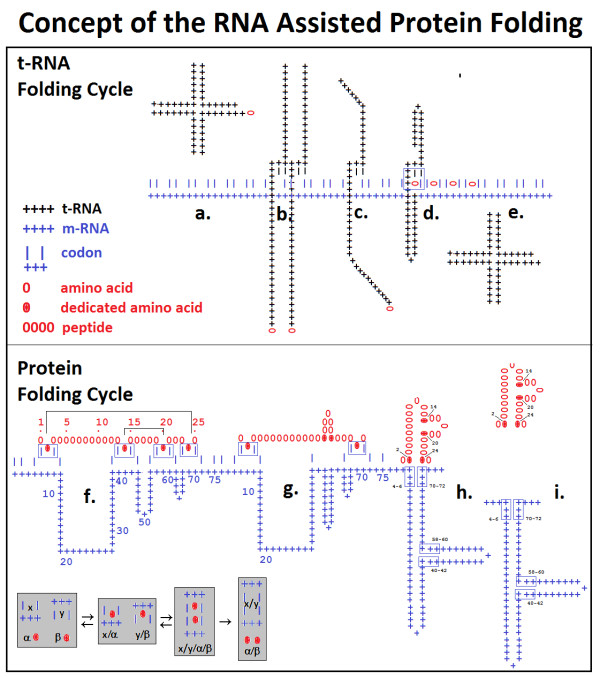
**Concept of RNA-assisted protein folding**. The model comprises tRNA (upper part) and protein (lower part) folding cycles. During the tRNA cycle the aminoacyl -tRNA (clover-leaf form, (a)) unfolds, interacts with its codon and the previously attached tRNA (b), refolds to a configuration that brings the amino acid tail into close proximity with the codon-anticodon site (c, d), loses the amino acid, refolds to its original cloverleaf configuration (e) and is recycled. The protein folding cycle starts when the peptide synthetase forms peptide bonds between individual amino acids. Some "dedicated" amino acids remain attached to their codons, but most are displaced. The difference in length between the peptide and mRNA creates mRNA folds (f) and the interaction between complementary codons creates peptide folds (g) one after the other (h). The growing peptide-mRNA complex dissociates after "pairing" the last "dedicated" amino acid pair with its corresponding codon pair (i) and the mRNA is recycled. The numbers indicate the positions of the dedicated amino acids and their codons in a 25 amino acid-long peptide and its 75 nucleotide-long mRNA. The inserted gray boxes depict the rules of the Proteomic Code [2]: co-locating amino acids (α and β) are coded by codons (x and y) which are complementary to each other at the 1st and 3rd nucleotide positions, they form different complexes with each other (x/α, y/β, x/y/α/β, x/y, α/β).

The tRNA cycle has significance for understanding the Proteomic Code [1] and Nucleic Acid-Assisted Protein Folding [2]. The 30-year-old concept of the Proteomic Code suggests that adjacent amino acids in a protein structure are encoded by partially complementary codons, where the 1st and 3rd codon residues are encoded by complementary nucleic acids in reverse orientation, while the second codon residues may be, but are not necessarily, complementary to each other. For a review and compilation of articles see [51]. Consequently, nucleic acids (tRNAs and mRNA together) form a 3D structure similar to the 3D structure of the encoded proteins, i.e. nucleic acids provide a kind of mold for the protein structures. This is the concept of nucleic acid chaperons [1]. The transfer of 3D information from mRNA to protein requires that the mRNA remains in physical contact with the peptide it encodes after translation. However, it is difficult to explain how this contact is possible when the involvement of the mediator, the tRNA, seemingly prevents any direct contact between these molecules. The tRNA cycle may provide an explanation: the involvement of tRNA in the information transfer from mRNA to peptide not only permits, but even requires, direct physical contact between the nucleic acid template and the encoded peptide (Figure 8).

The possibility of specific, high affinity interactions between codons and encoded amino acids has been the subject of long and intense debate, with a great many personalities involved. Francis Crick vehemently rejected this connection and stated that any connection between codons and encoded amino acids is only the result of a "frozen accident" [7]. His view, owing to his strong personality and his Nobel Prize in 1962, has been and remains very successful even, and affects the perceptions of the scientific community on this question. On the other hand, there is very strong evidence to suggest that amino acids are evolutionarily strongly connected to their codons [7,51-53], and they preferentially collocate with each other in protein-nucleic acid complexes [54]. We find that specific codon-amino acid interactions are necessary, at least for some "dedicated" examples that mark structurally critical points in the peptide and mRNA sequences and perform the correct protein folding under the guidance of a nucleic acid. The literature [55], as well as our studies [54], indicates that Arg, Lys, Asn and Gln are examples of these "dedicated" amino acids.

## Conclusions

We suggest that tRNAs have a far more complex role in translation than functioning only as passive adaptors. They are able to change form and interact with each other, and this change seems to be necessary for mRNA-assisted protein folding to be carried out. These new roles explain the large size and generous redundancy of tRNAs.
